# Mitochondrial function and oxidative stress markers in higher-frequency episodic migraine

**DOI:** 10.1038/s41598-021-84102-2

**Published:** 2021-02-25

**Authors:** Elena C. Gross, Niveditha Putananickal, Anna-Lena Orsini, Deborah R. Vogt, Peter S. Sandor, Jean Schoenen, Dirk Fischer

**Affiliations:** 1 Division of Paediatric Neurology, University Children’s Hospital Basel (UKBB), University of Basel, Basel, Switzerland; 2 Neurology Department, University Hospital Basel (USB), University of Basel, Basel, Switzerland; 3 Clinical Trail Unit (CTU), Department of Clinical Research, University Hospital Basel (USB), University of Basel, Basel, Switzerland; 4 RehaClinic Group, Bad Zurzach, University of Zurich, Zurich, Switzerland; 5 University of Zurich, Zurich, Switzerland; 6 Headache Research Unit, Dept of Neurology-Citadelle Hospital., University of Liège, Liège, Belgium

**Keywords:** Migraine, Diagnostic markers, Endocrine system and metabolic diseases

## Abstract

Increasing evidence points towards the role of mitochondrial functioning, energy metabolism, and oxidative stress in migraine. However not all previous research has been conclusive and some mitochondrial function/oxidative stress markers have not yet been examined. To this end, alpha-lipoic acid (ALA), total thiols, total plasma antioxidant capacity (TAC), lipid peroxide (PerOx), oxidised LDL (oxLDL), HbA1c and lactate were determined in the serum of 32 higher frequency episodic migraineurs (5–14 migraine days/ months, 19 with aura, 28 females) in this cross-sectional study. The majority of patients had abnormally low ALA and lactate levels (87.5% and 78.1%, respectively). 46.9% of the patients had abnormally high PerOx values, while for thiols and TAC over one third of patients had abnormally low values (31.2% and 37.5%, respectively). 21.9% of patients had abnormally low HbA1c and none had an HbA1c level above 5.6%. oxLDL was normal in all but one patient. This study provides further evidence for a role of oxidative stress and altered metabolism in migraine pathophysiology, which might represent a suitable therapeutic target. ALA, being too low in almost 90% of patients, might represent a potential biomarker for migraine. Further research is needed to replicate these results, in particular a comparison with a control group.

This study is part of the trial registration: ClinicalTrials.gov: NCT03132233, registered on 27.04.2017, https://clinicaltrials.gov/ct2/show/NCT03132233.

## Introduction

Migraine is a complex, common and debilitating neurological disorder^1^ and yet its primary pathogenic mechanisms are not completely understood. Despite being referred to as a “hypoglycemic headache” in 1935 already^2^, the focus of clinical and basic research has shifted towards (neuro-)vasculature, cerebral excitability and neurotransmission for several decades. In recent years, metabolism and mitochondrial (dys-)function have regained interest and various lines of evidence—much of it clinical—are suggesting that migraine is—at least partially—an energy deficit syndrome of the brain.

For example, magnetic resonance spectroscopy (MRS) studies in migraine consistently show abnormalities of mitochondrial oxidative phosphorylation (OXPHOS), such as hypometabolism or decreased ATP levels^3–13^. These findings are supported by early studies showing metabolic changes induced by fasting, glucose or insulin administration, which can even trigger migraine attacks in susceptible patients^14–20^.

Further support for a link to energy metabolism and/or mitochondrial functioning comes from the migraine preventative effect of several nutraceuticals^21^, such as riboflavin at high dose (200–400 mg/ day)^22–28^; coenzyme Q10 (400 mgcapsulesor300 mgliquidsuspension)^29–34^, magnesium^35^ and alpha-lipoic acid (ALA; 600 mg)^36–38^. Dietary approaches, such as a ketogenic diet (KD), which promotes the hepatic production of an alternative energy substrate for the brain and to some extent mimics the state of fasting, have been shown to be migraine protective^39–44^ (see^45^ for potential mechanism of ketosis in migraine). Moreover, elevated plasma lactate and pyruvate levels have been reported, but mostly in severely affected patients, such as migrainous stroke^46,47^.

Reactive oxygen species (ROS), such as hydroxyl radicals, hydrogen peroxide and superoxide radical anions, are produced as by-products of normal metabolic processes, such as electron transport in mitochondria, host defense or enzymatic reactions^48^. In healthy organisms, antioxidant defense systems protect the cells and tissues against these species^49,50^. When the generation of ROS exceeds the body’s antioxidant capacity, oxidative stress, i.e. damage to cellular constituents, such as proteins, lipids, DNA and sugars, occurs^48,50^. Oxidative stress could be the common denominator of most migraine trigger and aggravating factors^51^. While for some of the more “metabolic” triggers, such as fasting /skipping a meal, physical exercise, stress and relaxation thereafter a direct link to energy homeostasis seems obvious, most of the seemingly unrelated triggers, such as ovarian hormone changes, weather changes, alcohol, strong odors, intense light and loud noises, also have a potential common denominator: changes in mitochondrial metabolism and/or oxidative stress (see reviews^51,52^ for further details). Mechanistically, transient receptor potential (TRP) channels, expressed in meningeal nociceptive nerve terminals, can be activated by oxidative, nitrosative and electrophilic stress^53,54^, thereby providing a mechanism by which known migraine trigger factors that increase oxidative stress could lead to migraine pain.

Increased oxidative/nitrosative stress and/or decreased anti-oxidant capacity have directly been found in migraine patients^55–68^, however the results were not always consistent. Of all biomarkers examined, superoxide dismutase activity seemed to be the one consistently reduced in migraine patients, also interictally^69^. The inconsistent results for the other markers could be due to differences in methodology, patient selection and variations depending on the migraine cycle. Regarding the latter, nitrosative and oxidative stress^68^ and nitric oxide^69^ were significantly elevated during migraine attacks, but not interictally. Whether those metabolic abnormalities observed are primary or secondary to the disease remains to be determined.

The aim of this study was to analyse peripheral markers of mitochondrial functioning/energy metabolism that have either not previously been looked at (to the best of our knowledge) or previously produced inconsistent results, in order to further decipher the metabolic face of migraine, with a particular focus on oxidative stress markers. Through the measurement of ALA, thiols, total plasma antioxidant capacity (TAC), lipid peroxide (PerOx), oxidised LDL (oxLDL), HbA1c and lactate in the serum of 32 medium to high frequency migraineurs the present study aimed to provide peripheral markers of mitochondrial functioning / energy metabolism that could easily be analysed by most practitioners to potentially assist with individualised treatment. Taking into account previous findings, we hypothesised that the three-month average plasma glucose concentration (Hba1c) would be reduced, the oxidative stress markers ALA, thiols and TAC would be reduced, PerPx and oxLDL would be increased and that there would be no difference in lactate levels in our episodic migraine patient population.

## Materials and methods

### Patients

After receiving ethical approval from the local ethics committee (Ethikkommission Nordwest-und Zentralschweiz (EKNZ), PB 2016-00497), written informed consent was obtained from each patient. We recruited patients from the Neurology Out-patient Departments at the University Hospitals of Basel, Bern, Zurich, the University of Basel, using internet announcements and by advertising in local busses and trains. Patients were part of the MigraKet trial^70^ (ClinicalTrials.gov Identifier: NCT03132233). The diagnosis of migraine was made by a trained neurologist based on the criteria according to the International Headache Society^71^. A German version of the migraine disability assessment (MIDAS) was used to assess migraine related disability^72^. All experiments and methods were performed in accordance with relevant guidelines and regulations (EKNZ, PB 2016-00497).

#### Inclusion criteria

Patients were included, if they were previously diagnosed with migraine (with or without aura) in accordance with the ICHD-3 (International Classification of Headache Disorders version 3 Beta) Classification criteria^71^, were between the ages of 18 and 65 years, experienced between 5 and 14 migraine days per month (over the last 4 months), had an age of onset of migraine less than 50 years old and had not changed the type, dosage or frequency of any prophylactic medication (exclusive of medications taken for acute relief of migraine symptoms) for at least 3 months prior to study onset.

#### Exclusion criteria

Patients were excluded, if they had a history of any significant neurological, psychiatric or other medical condition or a known history of suspected secondary headache, if they were taking simple analgesics or non-steroidal anti-inflammatory drugs (NSAIDs) more than 14 days per 4 weeks or triptans on more than 10 days per 4 weeks for headaches or other body pain or any prescription opioids, if they had a previous diagnosis of medication overuse headache, which has reverted to episodic migraine within the last 6 months or met ICHD-3 Beta Classification criteria^71^ for chronic migraine (> 15 headache days per month), if they had had a surgery for migraine prevention, if they had received botulinum toxin injections within the last 6 months or if they were pregnant.

### Laboratory procedures

The following mitochondrial function markers were examined (normative values from local laboratory (Ganzimmun Diagnostic AG, Mainz, Germany or University Hospital Basel, Basel Switzerland) indicated in brackets):

- Total anti-oxidative capacity (TAC; > 280 μmol/l)
- Oxidated LDL (OxLDL; < 235 ng/ml)
- Alpha-lipoic acid (ALA; > 0.52 μg/l)
- Lipid-peroxide (PerOx; < 180 μmol/l)
- Thiols (complete; > 55 μmol/l)
- HbA1c (4.8–5.9%)
- Lactate (1.1–2.0 mol/l)

Venous blood samples were drawn from an antebrachial vein following overnight fasting. After 30–60 min at room temperature the serum was separated from the rest of the blood by centrifugation at 1300G for 10 min. Aliquots of serum were stored at − 80 °C. One aliquot contained 0.3 ml serum. Three aliquots per patient were sent for analysis. Blood samples for HbA1C and lactate were not stored, but immediately sent at room temperature to the inhouse laboratory for immediate analysis.

#### Total antioxidant capacity

TAC was measured using an ImAnOx‐assay (Ganzimmun Diagnostic AG, Mainz, Germany) (inter-assay variation: 2.43%; intra‐assay variation: 2.33%). This photometric test reflects the sum of all antioxidant components by measuring hydrogen peroxide (H_2_O_2_) degradation by the serum antioxidants. Please refer to^73^ for further details.

#### Peroxides

Serum total peroxide concentrations were determined photometrically by the peroxide concentration assay (Ganzimmun Diagnostic AG, Mainz, Germany) (inter-assay variation: 3.5–3.6%; intra‐assay variation: 2.0–3.6%), which is based on the reaction of horseradish peroxidase with plasma peroxides using tetramethylbenzidine as a chromogen substrate (450-nm wavelength). Please refer to^74^ for further details.

#### Oxidised LDL

The measurement of serum oxidised LDL was performed using a sandwich ELISA method (ox-LDL ELISA kit, Ganzimmun Diagnostic AG, Mainz, Germany) (inter-assay variation: 9–11%; intra‐assay variation: 3.9–5.7%). No antioxidants were added to the plasma samples before collection. Please refer to^75^ for further details.

#### Thiols

Thiols were determined in serum using the ImmuChrom HPLC assay (Ganzimmun Diagnostic AG, Mainz, Germany) (inter-assay variation: 5.5–5.9%; intra‐assay variation: 5.2–5.8%), where thiols present in serum proteins are precipitated with 80% saturated ammonium sulfate. Please refer to^76^ for further details.

#### Alpha-lipoic acid

ALA was determined in serum using the HPLC method (Ganzimmun Diagnostic AG, Mainz, Germany). The standards and the solutions were sourced from Merck KGaA. In brief, 100 µl of the serum sample was diluted in 1.9 ml acetone. The sample was mixed thoroughly for 5 s. After that, the sample was centrifuged for 10 min at 3500 U/min. 800 µl of the supernatant was evaporated under air at 45 °C for 10 min. The dry residue was dissolved in 400 µl 30/70 0.1% acetic acid/ acetone mix. The sample was mixed thoroughly for 5 s. 300 µl of the solution was transferred in a HPLC vial. A calibration curve in empty serum was prepared with five different standard concentrations. The highest standard was 200 µg/l and the lowest 12.5 µg/l. The preparation of the standard was equal to the sample preparation. The concentration of ALA was determent by LC–MS/MS with a Varian 320 in negative mode. For the HPLC method an Atlantis T3, 3 µm, 150 × 2.1 mm column from Waters GmbH was used. The isocratic gradient was 30% 0.1% acetic acid and 70% acetone with a flowrate of 0.3 ml/min and an injection volume of 40 µl. The runtime was 4 min with a retention time of ALA at 2.1 min.

#### Glycosylated hemoglobin (HbA1c) and lactate

HbA1c and lactate were analysed using the Cobas 8000 c502 (Roche Diagnostics) at the laboratory of the University Hospital Basel. HbA1c was determined using the HbA1c Turbidimetric Immunoassay (Tina-quant, 3rd generation) from haemolysed full blood EDTA samples. Lactate was analysed by centrifugation at 3004G for 8 min of blood EDTA / fluorid samples, plasma was then separated. Following this, plasma samples were immediately analysed using an enzymatic colour assay.

### Statistical analyses

Summary statistics (mean, median, interquartile range, minimum and maximum) and the number and percentage of patients with non-normal values are indicated for each biomarker. Individual measures are both shown separately in the original units and on a common, standardized scale for better comparison. For endpoints with normal ranges (HbA1c and lactate), the original values were scaled such that the minimum and maximum of the normal range correspond to 0 and 1. Values < 0 are thus abnormally low and values > 1 abnormally high. For the other endpoints with a single normal cut-off, the original values were centered, such that the cut-off corresponds to 0, and scaled by dividing by the standard deviation, such that 1 corresponds to one standard deviation. For TAC, ALA and thiols, values < 0 are considered abnormal, while for oxLDL and PerOx, values > 0 are considered abnormal. Boxplots were drawn as follows. The Boxes contain the 25% through 75% quantiles (spanning the interquartile range), the thick horizontal line is the median. Whiskers indicate the most extreme values lying within the box-edge and 1.5 * the interquartile range. All eventual further values (outliers) are plotted as individual points.

Several post-hoc analyses were performed. Correlations of biomarkers and migraine intensity were examined visually and Spearman’s rank correlation coefficient was calculated. As measure of migraine intensity, the number of migraine days and the MIDAS score at baseline were considered. Subgroup comparisons were performed between patients with and without migraine prophylaxis, between patients with and without acute migraine attack at baseline ± 2 days and between MA and MO. Subgroups were tested for a difference using Wilcoxon’s rank sum test (continuous outcomes) and Fisher’s exact test (frequencies).

In accordance with the exploratory nature of the analyses, *p* values should not be interpreted as confirmative, but can be useful in identifying hypotheses worth of further investigation. In accordance with recent statistical guidelines, the term ‘statistically significant’ is not used (following the strong suggestions made in the ASA Editorial on ‘Moving to a world beyond p < 0.05’^77^).All analyses were conducted using the statistical software package R^78^.

## Results

### Study population

Thirty-two patients were included in the study^4,27^. The mean age was 34 ± 10.8 years. Twelve patients had migraine without aura (MO) and 20 migraine with aura (MA). Patient characteristics and demographics’ information are shown in Table 1. Eleven patients were using at least one stable migraine prophylaxis (no changes within at least 3 months prior to study onset) (see Table 2 for the migraine preventatives used).

**Table 1:** Summary statistics of patient characteristics.

Variables	All patients
Age, mean (SD)	34 (10.8)
Female, N (%)	28 (87.5)
Male, N (%)	4 (12.5)
Height (m), mean (SD)	1.7 (01.)
Weight (kg), mean (SD)	67.3 (13.9)
Migraine days / months, mean (SD)	8.6 (2.1)
Migraine without aura, N (%)	12 (37.5)
Migraine with aura, N (%)	20 (62.5)
MIDAS, mean (SD)	31 (19.9)
Stable migraine prophylaxis, N (%)	11 (34.4)
No migraine prophylaxis, N (%)	21 (65.6)

Categorical variables are summarized as frequencies and percentages (%), numerical variables are summarized by mean and one standard deviation (sd).

SD = standard deviation, m = meter, kg = kilogram.

**Table 2:** Types and frequencies of migraine prophylactic treatments.

Type	N
Antidepressants	2
Anticonvulsants	2
Beta-blockers	5
Cefaly™ neurostimulator	1
Calciumantagonists	2
Magnesium	7
Riboflavin/Vitamin B2	1

Note that more than one type of prophylactic drug could be used.

### Summary statistics

**Figure 1:**
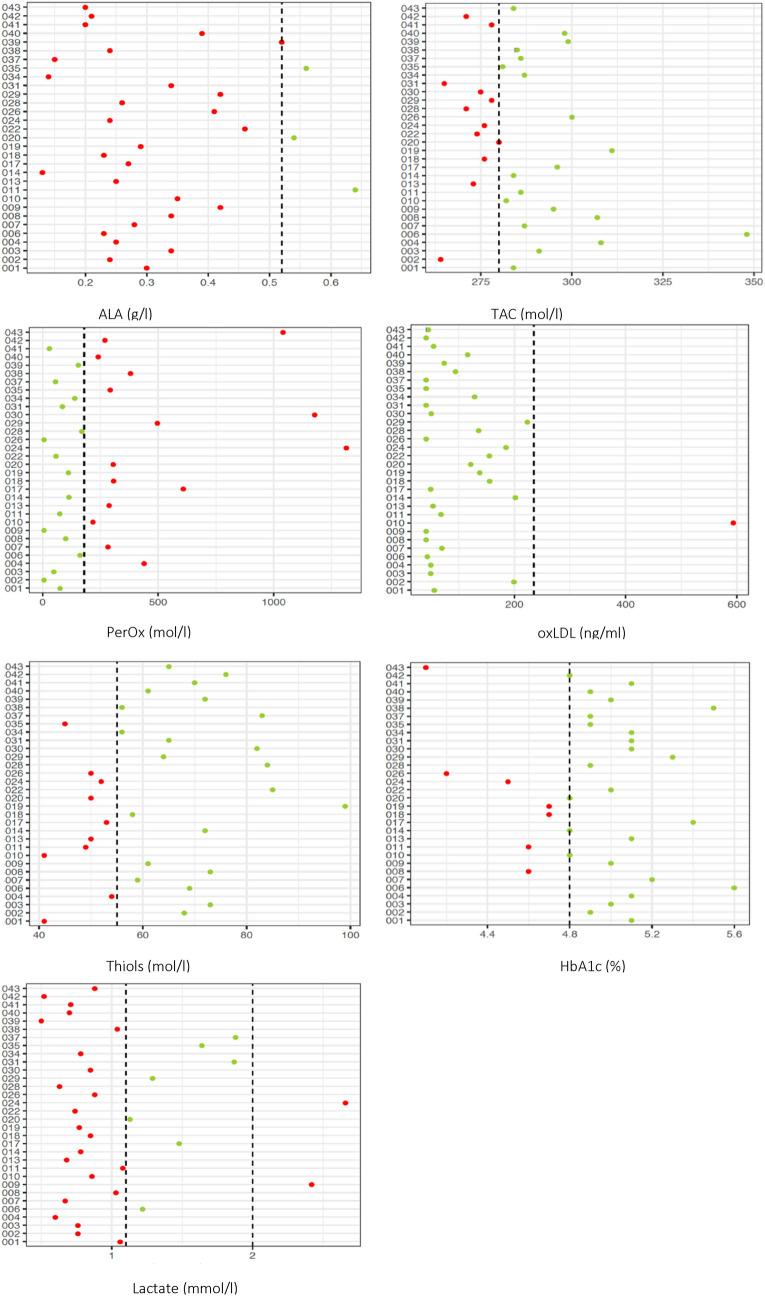
Baseline values of mitochondrial function markers for each patient. Dashed lines indicate the normal-range cut-off(s). Green dots indicate values within normal range, red dots indicate values outside the normal range. For alpha lipoic acid one very extreme value (13.25) has been removed for better visualisation. ALA = Alpha-lipoic acid; ox-LDL = oxidised LDL; PerOx = total lipid peroxide; TAC = total antioxidant capactity.

**Figure 2:**
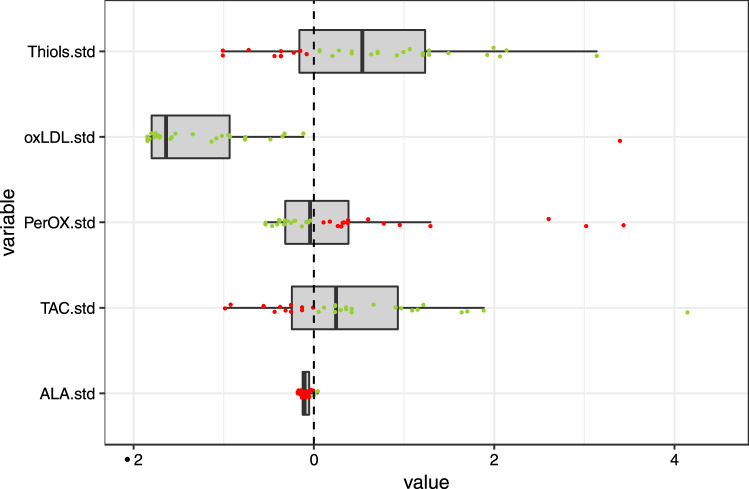
Standardized (= .std) baseline values of mitochondrial function markers with a single cut-off. Values are standardized such that zero (the dashed line) indicates the normal cut-off, 1 indicates one standard deviation, 2 indicates two standard deviations, etc. Green dots indicate values within normal range, red dots indicate values outside the normal range. For alpha lipoic acid one very extreme value (5.6) has been removed for better visualisation. ALA = Alpha-lipoic acid; ox-LDL = oxidised LDL; PerOx = total lipid peroxide; TAC = total antioxidant capactity.

**Figure 3:**
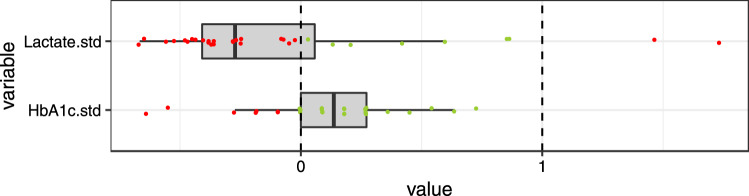
Standardized (= .std) baseline values of mitochondrial function markers with two cut-offs. Values are standardized such that zero (the dashed line) indicates the lower cut-off of the normal range and 1 indicates the upper cut-off of the normal range. Green dots indicate values within normal range, red dots indicate values outside the normal range. ALA = Alpha-lipoic acid; ox-LDL = oxidised LDL; PerOx = total lipid peroxide; TAC = total antioxidant capactity.

Summary statistics of all endpoints are given in Table 3. Single observations are visualized in their original units (Fig. 1) and scaled (Figs. 2, 3).

**Table 3:** Summary statistics of mitochondrial function markers.

Marker	Normal range	Minimum	Q1	Median	Mean	Q3	Maximum	Non-normal
ALA	> 0.52 μg/l	0.13	0.24	0.29	0.72	0.41	13.25	28 (87.5%)
TAC	> 280 μmol/l	264.0	276.00	284.00	286.88	295.25	348.00	12 (37.5%)
PerOx	< 180 μmol/l	6.0	75.50	166.00	283.31	306.50	1315.00	15 (46.9%)
Ox-LDL	< 235 ng/ml	41.3	45.10	62.15	106.35	136.38	593.90	1 (3.1%)
Thiols	> 55 μmol/l	41.0	52.75	62.50	63.62	72.25	99.00	10 (31.2%)
HbA1c	4.8–5.9%	4.1	4.80	4.95	4.93	5.10	5.60	7 (21.9%)
Lactate	1.1–2.0 mol/l	0.5	0.73	0.85	1.05	1.15	2.66	25 (78.1%)

Q1–Q3: interquartile range; non-normal: number (%) of patients with values outside normal range.

ALA = Alpha-lipoic acid; ox-LDL = oxidised LDL; PerOx = total lipid peroxide; TAC = total antioxidant capactity.

For ALA and lactate, the majority of patients had abnormally low values (28/32 (88%) and 23/32 (72%) respectively). Only two patients’ lactate levels were too high. For one patient, an extremely high level of ALA (13.25) was measured. For PerOx half of the patients (46.9%) had abnormally high values. For thiols and TAC about one third of patients had abnormally low values (31.2% and 37.5%, respectively). For HbA1c about 20% of patients (21.9%) had abnormally low values and no one had an HbA1c above 5.6%. For oxidated LDL, a very high, abnormal level was measured in one patient, while for all other patients, the levels were in the normal range.

### Correlations of mitochondrial function biomarkers and migraine severity

We found no indication for a correlation of the 7 mitochondrial function biomarkers with MIDAS score or number of migraine days per month at baseline. Corresponding correlation coefficients and *p* values are given in supplementary information section 1.1.

### Comparison between patients with and without migraine prophylaxis

Summary statistics of absolute levels of the mitochondrial function biomarkers and the frequencies of patients with abnormal values according to migraine prophylaxis are presented in supplementary information section 1.2. Our data provide no evidence for an effect of migraine prophylaxis.

### Comparison between patients studied during or outside of an attack

Summary statistics of absolute levels of the mitochondrial function biomarkers and the frequencies of patients with abnormal values according to acute migraine attack at baseline (baseline visit ± 2 days) are presented in supplementary information section 1.3. Most patients presented with acute migraine at baseline; for one patient this information is missing. Our data provide no evidence for any difference between these two groups.

### Comparison between patients with and without aura

Summary statistics of absolute levels of the mitochondrial function biomarkers and the frequencies of patients with abnormal values according to aura are presented in supplementary information section 1.4. We found no evidence for differences between patients with or without aura, neither in the absolute values of the biomarkers nor in the proportions of patients with abnormal values.

## Discussion

We have shown that apart from oxLDL and HbA1c most other markers of mitochondrial functioning showed abnormalities in a significant proportion (> 30%) of the patients examined.

### ALA

To the best of our knowledge ALA levels have not previously been determined in migraine. Almost 90% of patients in this sample had abnormally low values of ALA. ALA, also known as thioctic acid, is an eight-carbon, sulfur-containing compound that functions as a water- and fat-soluble antioxidant^79,80^. It can directly (by removing reactive species) and indirectly (by chelating transition metal ions) reduce oxidative stress^79,80^. The human body can synthesize small amounts of ALA^79^. ALA also plays an important role as co-enzyme in energy metabolism^79–81^. Furthermore, it is able to regenerate other antioxidants, such as vitamin C and E, CoQ10, it increases intracellular glutathione and activates endogenous antioxidant systems^82–84^. Apart from its anti-oxidant action, ALA seems to assist weight loss^85^, increase insulin sensitivity and decrease blood lipids^86^. All of these mechanisms are probably migraine relevant. Interestingly, ALA supplementation (300–600 mg) per day has been shown to significantly reduce migraine attack frequency, severity and duration^36–38^, which seems to align with our findings. Further research is needed to see, whether this finding is specific to our medium–high frequency episodic migraine population or a general characteristic of migraine or even a general characteristic of other (neurological) diseases with a mitochondrial/oxidative stress component. Additionally, as ALA measurements are less established and standardised than other markers, these results should be replicated in a larger cohort, with a different laboratory and using a control group. Should this finding be replicated and migraine specific, ALA might represent a potential biomarker.

### TAC

Serum (or plasma) concentrations of different antioxidants can be measured separately, but since the measurement of different antioxidant molecules individually is impractical and costly and their antioxidant effects are additive, the total antioxidant capacity of a sample is typically measured, and this is typically referred to as total antioxidant capacity (TAC), total antioxidant status (TAS) or other synonyms, which will be used interchangeably.

Almost 40% of our patients had abnormally low TAC being in line with results of previous research. A study on 75 MO patients demonstrated that the levels of total antioxidants were decreased and the levels of total oxidants and the oxidative stress index were increased^55^. Another study found TAC to be significantly reduced in migraineurs compared to controls^66^. TAC levels increased after successful prophylactic treatment compared to the baseline, irrespective of treatment modality (rTMS versus amitriptyline) and the increase correlated with treatment success^66^.We assume higher TAC with lower migraine severity, less recent oxidative stress exposure, and increased distance to previous and future migraine attack. These assumptions would have to be validated in future research.

### PerOx

Lipid peroxidation is the oxidative degradation of lipids via free radical damage of the lipids in cell membranes, polyunsaturated fatty acids in particular. The end products of lipid peroxidation are reactive aldehydes, such as 4-hydroxynonenal (HNE) and malondialdehyde (MDA). Free radicals cause increased accumulation of these lipid peroxidation by-products in the blood. About half of the patients had abnormally high total PerOx levels, being in line with previous research. Several studies have found serum levels of MDAs to be significantly elevated in migraine patients^56,67^, even in the interictal phase^87^.

### oxLDL

Oxidized low-density lipoprotein (LDL) is a harmful type of cholesterol that is produced when normal LDL cholesterol is damaged by chemical interactions with ROS. All but one patient had normal levels for oxLDL, which is in contrast to the study of Bernecker et al. that found highly significantly elevated levels oxLDL in female migraineurs^57^. This result could be due to differences in study population, as the migraineurs of the Berecker et al. study tended to have metabolic syndrome and had generally higher BMIs as our migraine patient population.

### Thiols

The term “thiol” refers to organic compounds containing sulfur (in form of the functional group –SH, the thiol group). Thiol groups are able to destroy ROS and other free radicals by enzymatic as well as nonenzymatic mechanisms^88^. Total thiol levels have previously been used to evaluate excess free radical generation, both in physiological and pathological conditions^89^. Protein thiol levels in serum have been shown to be a direct measure of the in vivo reduction/oxidation (redox) status in humans, because thiols react readily with ROS to form disulfides^76^. Thiol redox homeostasis plays an important role in neurogenerative diseases^90^ and in nine other categories of human disorders serum protein thiols have been found to be significantly reduced compared to healthy controls^76^.

About one third of patients had abnormally low serum thiol levels, but this seems to be in line with previous research. A larger study found significantly reduced thiol levels in 151 migraine patients (74 MO, 77 MA) compared to 70 healthy controls and there was a negative correlation with migraine disability^61^. A negative correlation between the levels of total thiols and the duration of the headaches has also been demonstrated^55^. However, others studies found no significant difference in thiol groups between patients and controls, even during attacks^68^ and one study even found higher total (–SH + – S – S–) & native thiol (–SH) levels in serum of migraineurs, but this did not correlated with disease severity or migraine type^63^. Recent exposure to oxidative stress, migraine severity, time in the migraine cycle and similar aspects could explain the different results.

### HbA1c

HbA1c (glycated hemoglobin) is an indication of the average blood glucose levels over the last two to three months. Just over 20% of patients had abnormally low HbA1c levels and none of them had HbA1c levels that were above 5.6%. To the best of our knowledge HbA1c has rarely been looked at in migraine. One study found no significant difference in HbA1c levels between CM, EM and healthy controls^19^.

However, magnetic resonance spectroscopy (MRS) studies in migraine have consistently shown abnormalities of mitochondrial oxidative phosphorylation (OXPHOS), such as hypometabolism between^3–9^ and during migraine attacks^10^, in the resting brain and in the muscle following exercise^3,11,12^. A 16% decrease of absolute ATP levels in migraine without aura patients was also demonstrated interictally using 31P-MRS^13^. These findings are supported by early studies showing that metabolic changes induced by fasting, glucose or insulin administration can trigger migraine attacks; e.g. a 50 g glucose tolerance test (GTT) after a 10-h fast triggered a migraine in 6 out of 10 migraine patients reporting attacks associated with fasting^14^. Abnormal metabolic responses were also reported in GTT studies^14,15^ and interictal impaired glucose tolerance and insulin resistance has been reported in various other studies^16–20^. While only 20% of our migraineurs had abnormally low HbA1c levels, all levels tended to be on the lower side, despite reported higher carbohydrate diets. As HbA1c levels correspond to an average blood sugar measurement, low average values despite probable highs after carbohydrate rich meals could be an indication that there might be lows as well. This would be in line with previous neuroimaging and GTT research results, but it is speculation only and these assumption need to be confirmed by future research.

### Lactate

Lactate is typically measured to assess tissue oxygenation, arising from either decreased oxygen delivery or a disorder in oxygen use, both of which lead to increased anaerobic metabolism and increases in lactate levels. In certain types of migraine, especially migrainous stroke, elevated serum lactate and pyruvate levels have previously been reported^46,47^. In contrast to this, only 2 patients had abnormally high serum lactate levels in our cohort and over 70% of patients serum lactate levels were abnormally low.

While there is little data on serum lactate levels in migraine, data on brain lactate analysed with 1H-MRS have also been shown to vary due to patient selection (see review by Reyngoudt et al. (2012) for details^8^). Elevated brain lactate levels were found in some studies of MA^91,92^, but not in MO^93–96^. Occipital baseline lactate levels were increased in patients with visual auras, but not in those having complex neurological auras. By contrast, during photic stimulation lactate increased significantly in the latter, but not in the former^91^. Stimulus-induced lactate increases are physiological^97^ and can be explained by the neuron-astrocyte lactate shuttle^98^. Hence, their absence in migraine patients, whose neuronal activation is energetically more demanding^99^, could be considered pathological and might be contributing to an energetic crisis.

To the best of our knowledge, no recent studies have looked at baseline serum lactate levels in episodic migraine patients or subgroups thereof. More research is needed to replicate this finding; in particular a study combining lactate level quantification in the cortex with that of the periphery and with brain energetics seems warranted. We can only speculate as to why lactate levels were predominantly low in the majority of our patients. They all came rested, but fasted overnight to the trial site. Decreased baseline lactate levels might be a sign of increased cerebral lactate consumption and an indicator of an increased cerebral energy demand of the migraine brain, as in addition to ketone bodies, lactate constitutes the only other major alternative brain energy substrate from glucose and is used especially during times of high metabolic demands or hypoglycemia^100^. A study using 13C-L-lactate and magnetic resonance spectroscopy suggested that the contribution of plasma lactate to brain metabolism can be up to 60%^101^, which is very similar to ketone bodies. It could also be a sign of decreased lactate synthesis as demonstrated with 1H-MRS^91^.

In summary, we have shown that apart from oxLDL and HbA1c most other markers of mitochondrial functioning are abnormal in at least > 30% of the patients examined. As oxidative stress is a complex mechanism including different sources of ROS and various pathways, differing results in previous research may at least be partially caused by different oxidative stress parameters examined, e.g. MDA versus HNE, as well as by different study groups investigated, e.g. adults versus children, MA versus MO, females versus males, and differences in migraine severity, recent oxidative stress exposure and the time within the individual migraine cycle, where measurements were taken. Genetic research examining oxidative stress related genes in larger homogenous migraine cohorts could be interesting future research that would hardly be influenced by these factors.

Our data provide no evidence for correlations between any of the seven mitochondrial function / oxidative stress markers and migraine severity. This could be due to our sample population being fairly homogenous or the sample size being too small. In addition, we found no evidence for an effect of migraine prophylaxis. This is not surprising, since patients were still suffering from a substantial number of migraine days/months despite the prophylactic treatment (5–14 days/months), suggesting that the critical migraine pathophysiological mechanisms remained active. Furthermore, no evidence for an effect of a preceding or subsequent migraine attack has been found. This might be due to only 5 patients being migraine attack free within 2 days before and after the venous puncture, making an analysis of the potential impact of an attack difficult. We also found no evidence for a difference between MA and MO patients. For a randomly selected migraine cohort mainly recruited via public advertisements, the number of MA patients was unusually high (62.5%) in our study population. We can only speculate as to why this might be the case. Since participants were part of the 9 months MigraKet intervention trial^70^, it seems plausible that MA patients might have been more motivated to take place in such a lengthy trial and this led to the observed over-representation.

While we found no correlation between these mitochondrial function/oxidative stress markers and disease severity, differences in methodologies used and patient characteristics, recent oxidative stress exposure and also time in the respective migraine cycle is likely to play a role. Future research examining these markers at different time points during the migraine cycle and in different migraine types would be interesting.

The most important limitation of this study is the absence of a matched control group. While abnormally low levels in 90% of patients in the case of ALA are likely to be of importance, we cannot be sure that PerOx, TAC and thiol level findings would have been significantly different from controls. Future research is needed to replicate these findings in the presence of a control group. Secondly, the sample size was fairly small, in particular with regards to the correlation analyses. In addition, one third of patients was using a migraine prophylaxis. While our data provide no evidence for an effect of migraine prophylaxis, the inclusion of patients who are using a prophylaxis is not ideal.

## Conclusion

In conclusion, this study provides further support for metabolic abnormalities in migraine, in particular the role of increase oxidative stress and decreased antioxidant capacity respectively in migraine pathophysiology. The peripheral markers assessed here could easily be examined in most doctor’s offices and might assist personalised migraine treatment that targets oxidative stress and mitochondrial functioning; however, further research is needed to replicate these findings, ideally in the presence of a control group.

## Supplementary Information

Supplementary Information

## Data Availability

For inspection purposes, insight to the original data will be permitted to the members of the appropriate authorities and also for the members of the local ethics committee, EKNZ.

## Abbreviations

ALA: Alpha-lipoic acid
ATP: Adenosine triphosphate
CGRP: Calcitonin gene-related peptide
CM: Chronic migraineurs
EM: Episodic migraineurs
HbA1c: Glycosylated hemoglobin
ICHD-3: International classification of headache disorders version 3
IQR: Interquartile range
KD: Ketogenic diet
PerOx: Lipid peroxide
MA: Migraine with aura
MDA: Malondialdehyde
MIDAS: Migraine disability assessment
MO: Migraine without aura
NSAIDs: Non-steroidal anti-inflammatory drugs
oxLDL: Oxidised LDL
H-MRS: Proton magnetic resonance spectroscopy
H_2_O_2_: Hydrogen peroxide
HNE: 4-Hydroxynonenal
OXPHOS: Oxidative phosphorylation
ROS: Reactive oxygen species
SOD2: Superoxide dismutase 2
TAC: Total plasma antioxidant capacity
TRP: Transient receptor potential channels
