# Supplementary material for: Mitochondrial function and oxidative stress markers in higher-frequency episodic migraine
Source: Sci Rep. 2021 Feb 25;11:4543. doi: 10.1038/s41598-021-84102-2 (PMC7907128; doi:10.1038/s41598-021-84102-2)
Supplement: Supplementary file 1 — Supplementary Information [file 41598_2021_84102_MOESM1_ESM.docx]

Research Article

Mitochondrial function and oxidative stress markers in higher-frequency episodic migraine

Elena C. Gross ^1^,* Niveditha Putananickal ^2^, Anna-Lena Orsini^3^, Deborah R. Vogt^4^, Peter S. Sandor ^5,6^, Jean Schoenen ^7^, and Dirk Fischer ^8^

^1^ Division of Paediatric Neurology, University Children’s Hospital Basel (UKBB), University of Basel, Switzerland; elena.gross@oxfordalumni.org

2 Division of Paediatric Neurology, University Children’s Hospital Basel (UKBB), University of Basel, Switzerland; [Niveditha.Putananickal@ukbb.ch](mailto:Niveditha.Putananickal@ukbb.ch)

3 Division of Paediatric Neurology, University Children’s Hospital Basel (UKBB) & Neurology Department, University Hospital Basel (USB),, University of Basel, Switzerland; [Anna-Lena.Orsini@usb.ch](mailto:Anna-Lena.Orsini@usb.ch)

4 Clinical Trail Unit (CTU), Department of Clinical Research, University Hospital Basel (USB), University of Basel, Switzerland; [deborah.vogt@usb.ch](mailto:deborah.vogt@usb.ch)

5 RehaClinic group, Bad Zurzach; University of Zurich, Switzerland; [p.sandor@rehaclinic.ch](mailto:p.sandor@rehaclinic.ch)

6 University of Zurich, Switzerland

7 Headache Research Unit. University of Liège. Dept of Neurology-Citadelle Hospital. Liège. Belgium; jschoenen@uliege.be

8 Division of Paediatric Neurology, University Children’s Hospital Basel (UKBB), University of Basel, Switzerland, [dirk.fischer@ukbb.ch](mailto:dirk.fischer@ukbb.ch)

***** Correspondence: [elena.gross@oxfordalumni.org](mailto:elena.gross@oxfordalumni.org)

Supplemental Information

1.1. Correlations of mitochondrial function biomarkers and migraine severity

We found no indication for a correlation of the 7 mitochondrial function biomarkers with MIDAS score or number of migraine days per month at baseline (Figures 1 and 2 and Table 1). Corresponding correlation coefficients and p-values are given in Table 4.

**Figure 1:** Mitochondrial function biomarkers vs MIDAS score at baseline.

Alpha lipoic acid: outlier data for one patient with an extremely high value (>10) is not shown. Smoothingcurves, using locally estimated scatterplot smoothing (LOESS) with span 1.0, are shown.

ALA = Alpha-lipoic acid; ox-LDL = oxidised LDL; PerOx= total lipid peroxide; TAC = total antioxidant capactity

**Figure 2:** Mitochondrial function biomarkers vs number of migraine days per month at baseline.

Alpha lipoic acid: outlier data for one patient with an extremely high value (>10) is not shown. Smoothing curves, using locally estimated scatterplot smoothing (LOESS) with span 1.0, are shown.

ALA = Alpha-lipoic acid; ox-LDL = oxidised LDL; PerOx= total lipid peroxide; TAC = total antioxidant capactity

**Table 1:** Correlations of mitochondrial function markers with migraine severity at baseline.

ϱ (rho) = Spearman’s rank correlation coefficient.

| Migraine intensity | Biomarker | ϱ (rho) | p-value |
| --- | --- | --- | --- |
| MIDAS score | ALA | -0.009 | 0.961 |
| MIDAS score | HbA1c | 0.05 | 0.787 |
| MIDAS score | Lactate | -0.13 | 0.491 |
| MIDAS score | oxLDL | 0.17 | 0.361 |
| MIDAS score | PerOX | 0.15 | 0.399 |
| MIDAS score | TAC | -0.0061 | 0.974 |
| MIDAS score | Thiols | -0.054 | 0.771 |
| Migraine days per month | ALA | 0.2 | 0.279 |
| Migraine days per month | HbA1c | 0.11 | 0.566 |
| Migraine days per month | Lactate | -0.25 | 0.163 |
| Migraine days per month | oxLDL | 0.22 | 0.228 |
| Migraine days per month | PerOX | -0.073 | 0.691 |
| Migraine days per month | TAC | -0.21 | 0.249 |
| Migraine days per month | Thiols | 0.28 | 0.127 |

ALA = Alpha-lipoic acid; MIDAS = migraine disability assessment; ox-LDL = oxidised LDL; PerOx= total lipid peroxide; TAC = total antioxidant capactity

1.2. Comparison between patients with and without migraine prophylaxis

Summary statistics of absolute levels of the mitochondrial function biomarkers and the frequencies of patients with abnormal values according to migraine prophylaxis are presented in Table 2. Our data provide no evidence for an effect of migraine prophylaxis.

**Table 2:** Comparison of mitochondrial function markers between patients with migraine prophylaxis and without.

| Marker | With prophylaxis (n=11) | No prophylaxis (n=21) | p |
| --- | --- | --- | --- |
| ABSOLUTE LEVELS: MEDIAN [IQR] | | | |
| ALA, median [IQR] | 0.28 [0.23, 0.44] | 0.29 [0.24, 0.39] | 0.858 |
| TAC, median [IQR] | 286.00 [278.00, 293.00] | 284.00 [276.00, 296.00] | 0.721 |
| PerOX, median [IQR] | 114.00 [52.50, 222.50] | 241.00 [100.00, 381.00] | 0.126 |
| oxLDL, median [IQR] | 74.00 [46.55, 170.10] | 54.90 [45.60, 128.50] | 0.498 |
| Thiols, median [IQR] | 69.00 [60.00, 72.50] | 58.00 [50.00, 70.00] | 0.159 |
| HbA1c, median [IQR] | 5.00 [4.85, 5.00] | 4.90 [4.70, 5.10] | 0.920 |
| Lactate, median [IQR] | 0.78 [0.75, 1.55] | 0.86 [0.71, 1.06] | 0.691 |
| NUMBER (%) OF PATIENTS WITH ABNORMAL VALUES | | | |
| ALA.abnorm, N (%) | 10 (90.9) | 18 (85.7) | 1.000 |
| TAC.abnorm, N (%) | 4 (36.4) | 8 (38.1) | 1.000 |
| PerOX.abnorm, N (%) | 3 (27.3) | 12 (57.1) | 0.217 |
| oxLDL.abnorm, N (%) | 0 ( 0.0) | 1 ( 4.8) | 1.000 |
| Thiols.abnorm, N (%) | 2 (18.2) | 8 (38.1) | 0.452 |
| HbA1c.abnorm, N (%) | 1 ( 9.1) | 6 (28.6) | 0.415 |
| Lactate.abnorm, N (%) | 8 (72.7) | 17 (81.0) | 0.933 |

.aborm = abnormal value (outside of the normal range); ALA = Alpha-lipoic acid; IQR = interquartile range; ox-LDL = oxidised LDL; PerOx= total lipid peroxide; TAC = total antioxidant capactity

1.3. Comparison between patients studied during or outside of an attack

Summary statistics of absolute levels of the mitochondrial function biomarkers and the frequencies of patients with abnormal values according to acute migraine attack at baseline (baseline visit ±2 days) are presented in Table 3. Most patients presented with acute migraine at baseline; for one patient this information is missing. Our data provide no evidence for any difference between these two groups.

**Table 3:** Comparison of mitochondrial function markers between patients with and without acute migraine at baseline (±2 days).

|  | Acute attack (n=26) | No attack (n=5) | p |
| --- | --- | --- | --- |
| ABSOLUTE LEVELS: MEDIAN [IQR] | | | |
| ALA, median [IQR] | 0.29 [0.23, 0.42] | 0.27 [0.24, 0.28] | 0.667 |
| TAC, median [IQR] | 283.00 [276.00, 297.25] | 286.00 [284.00, 287.00] | 0.914 |
| PerOX, median [IQR] | 194.00 [89.50, 307.50] | 114.00 [74.00, 283.00] | 0.554 |
| oxLDL, median [IQR] | 55.50 [41.88, 134.05] | 69.70 [68.20, 199.20] | 0.280 |
| Thiols, median [IQR] | 62.50 [52.50, 72.75] | 59.00 [53.00, 68.00] | 0.610 |
| HbA1c, median [IQR] | 4.95 [4.80, 5.10] | 4.90 [4.80, 5.20] | 0.828 |
| Lactate, median [IQR] | 0.87 [0.72, 1.20] | 0.78 [0.76, 1.08] | 0.809 |
| NUMBER (%) OF PATIENTS WITH ABNORMAL VALUES | | | |
| ALA.abnorm, N (%) | 23 (88.5) | 4 (80.0) | 1.000 |
| TAC.abnorm, N (%) | 11 (42.3) | 1 (20.0) | 0.662 |
| PerOX.abnorm, N (%) | 13 (50.0) | 2 (40.0) | 1.000 |
| oxLDL.abnorm, N (%) | 1 ( 3.8) | 0 ( 0.0) | 1.000 |
| Thiols.abnorm, N (%) | 8 (30.8) | 2 (40.0) | 1.000 |
| HbA1c.abnorm, N (%) | 6 (23.1) | 1 (20.0) | 1.000 |
| Lactate.abnorm, N (%) | 20 (76.9) | 4 (80.0) | 1.000 |

.aborm = abnormal value (outside of the normal range); ALA = Alpha-lipoic acid; IQR = interquartile range; ox-LDL = oxidised LDL; PerOx= total lipid peroxide; perTAC = total antioxidant capactity

1.4. Comparison between patients with and without aura

Summary statistics of absolute levels of the mitochondrial function biomarkers and the frequencies of patients with abnormal values according to aura are presented in Table 4. We found no evidence for differences between patients with or without aura, neither in the absolute values of the biomarkers nor in the proportions of patients with abnormal values.

**Table 4:** Comparison of mitochondrial function markers between patients with aura and those without.

|  | With aura (n=19 | Without aura (n=13) | p |
| --- | --- | --- | --- |
| ABSOLUTE LEVELS: MEDIAN [IQR] | | | |
| ALA, median [IQR] | 0.26 [0.21, 0.42] | 0.32 [0.26, 0.39] | 0.284 |
| TAC, median [IQR] | 284.50 [278.00, 292.00] | 281.00 [272.50, 298.25] | 0.471 |
| PerOX, median [IQR] | 150.50 [70.00, 395.50] | 194.00 [105.50, 284.25] | 0.876 |
| oxLDL, median [IQR] | 50.30 [43.03, 102.85] | 118.90 [65.73, 149.60] | 0.100 |
| Thiols, median [IQR] | 64.50 [55.50, 73.00] | 60.00 [50.00, 69.00] | 0.508 |
| HbA1c, median [IQR] | 5.00 [4.80, 5.10] | 4.90 [4.77, 5.03] | 0.223 |
| Lactate, median [IQR] | 0.26 [0.21, 0.42] | 0.32 [0.26, 0.39] | 0.284 |
| NUMBER (%) OF PATIENTS WITH ABNORMAL VALUES | | | |
| ALA.abnorm, N (%) | 17 (85.0) | 11 (91.7) | 1.000 |
| TAC.abnorm, N (%) | 6 (30.0) | 6 (50.0) | 0.451 |
| PerOX.abnorm, N (%) | 9 (45.0) | 6 (50.0) | 1.000 |
| oxLDL.abnorm, N (%) | 0 ( 0.0) | 1 ( 8.3) | 0.793 |
| Thiols.abnorm, N (%) | 5 (25.0) | 5 (41.7) | 0.555 |
| HbA1c.abnorm, N (%) | 4 (20.0) | 3 (25.0) | 1.000 |
| Lactate.abnorm, N (%) | 15 (75.0) | 10 (83.3) | 0.912 |

.aborm = abnormal value (outside of the normal range); ALA = Alpha-lipoic acid; IQR = interquartile range; ox-LDL = oxidised LDL; PerOx= total lipid peroxide; perTAC = total antioxidant capactity
